# Nupr1 regulates palmitate-induced apoptosis in human articular chondrocytes

**DOI:** 10.1042/BSR20181473

**Published:** 2019-02-15

**Authors:** Li Tan, Raghunatha R. Yammani

**Affiliations:** Section of Molecular Medicine, Department of Internal Medicine, Wake Forest School of Medicine, Winston-Salem, North Carolina, U.S.A.

**Keywords:** apoptosis, chondrocytes, nupr1, osteoarthritis, palmitate

## Abstract

Obesity, a major risk factor for the development of osteoarthritis (OA), is associated with increased circulating levels of free fatty acids (FFA). However, the role of these FFAs in OA pathophysiology is not clearly understood. In the present study, we found that palmitate treatment of human primary articular chondrocytes increased the expression of ER stress markers [activating transcription factor 4 (ATF4), C/EBP homologous protein (CHOP)] and apoptosis markers [cytochrome *c* and cleaved caspase-3 (CC3)]. Palmitate treatment also increased the expression of Nuclear protein 1 (Nupr1) and *tribbles* related protein 3 (TRB3), which are known negative regulators of cell survival pathways. Knockdown of Nupr1 or CHOP expression inhibited palmitate mediated increased expression of TRB3 and CC3, indicating that Nupr1 and CHOP cooperate to regulate cell survival and apoptotic pathways in human chondrocytes. *Nupr1* knockdown had no effect on CHOP expression whereas *CHOP* knockdown abolished the palmitate-mediated Nupr1 expression, indicating that CHOP is functional upstream to Nupr1 in this pathway. Moreover, overexpression of Nupr1 markedly increased the basal expression of pro-apoptotic molecules, including cytochrome *c* and CC3. Taken together, our study demonstrates that Nupr1 plays a crucial role in palmitate-induced apoptosis in human chondrocytes and Nupr1 as a potential novel drug target for the treatment of OA.

## Introduction

Osteoarthritis (OA) is the most common type of arthritis and one of the leading causes of global disability, affecting approximately 300 million people worldwide [1]. OA is characterized by progressive loss of articular cartilage due to abnormal anabolic and catabolic activities of chondrocytes [2,3]. Obesity, a major risk factor for OA [4], is associated with increased circulating levels of free fatty acids (FFA) [5]. Increased levels of FFA in synovial fluid and in the joint tissue are associated with increased severity of cartilage lesions in OA [6]. These studies clearly suggest that FFAs play an important role in OA pathophysiology. Accumulation of FFA in non-adipose tissue has been shown to induce inflammation and cell death [7]. We recently demonstrated that palmitate induced endoplasmic reticulum (ER) stress and activation of unfolded protein response (UPR) signaling pathway and promoted apoptosis in both meniscus and articular chondrocytes [8,9]. However, the molecular mechanisms underlying the effects of palmitate are still poorly understood.

C/EBP homologous protein (CHOP) is a multifunctional transcription factor that is activated during severe and chronic ER stress and is thought to play a major role in cell death [10,11]. Interestingly, recent studies have shown that ER stress also induces the expression of Nuclear protein 1 (Nupr1) and *tribbles* homolog related protein 3 (TRB3). Both of these proteins are known to play a major role in cell survival and apoptosis [12,13]. Nupr1 is a stress inducible pleiotropic transcriptional factor that plays major role in cellular physiology (regulating cell cycle, apoptosis, and autophagy) and in multiple human pathologies including cancer, diabetes, and cardiovascular diseases [12,14,15]. We recently demonstrated that Nupr1 is highly expressed in OA cartilage [16].

TRB3, a member of *tribble* homologous proteins, modulates signaling pathways associated with insulin resistance [17], IGF-1 [18], and cell survival [19,20], by inhibiting the phosphorylation/activation of the serine-threonine kinase Akt [17]. We have previously reported that TRB3 is expressed in OA cartilage and chondrocytes and that its expression level was increased during ER stress [18]. Furthermore, palmitate has been shown to induce TRB3 expression in podocytes [21] and liver cells [22]. Interestingly, recent studies have shown that Nupr1 plays an important role in CHOP-induced expression of TRB3 in response to ER stress in human tumor cells [23] and neuronal cells [24]. Moreover, CHOP directly up-regulates TRB3 transcription by binding a CHOP-binding site in the human *TRB3* promoter [13,25,26].

Given the association between ER stress and Nupr1, we hypothesize that Nupr1 is an important regulator of palmitate-induced apoptosis in human chondrocytes. In the present study, we investigated the role of Nupr1 during palmitate-induced ER stress to determine its role in OA pathogenesis. Our data are the first to show that palmitate induces Nupr1 expression in human chondrocytes, and that this protein is an important mediator of palmitate-induced apoptosis. We also demonstrated that overexpression of Nupr1 significantly induces caspase-mediated cell death in human chondrocytes.

## Materials and methods

### Reagents

Dulbecco’s modified Eagle’s medium /Ham’s F-12(1:1) (DMEMF), antibiotics, fetal bovine serum (FBS), poly-lysine, TRIzol reagent, DEPC-treated water, Lab-Tek chamber slide, and Micro BCA protein assay kit were purchased from Thermo Fisher Scientific. Pronase and Collagenase P were obtained from Roche Diagnostics. Phenylmethanesulfonyl fluoride solution (PMSF), phosphatase inhibitor cocktail 2, paraformaldehyde, fatty acid-free bovine serum albumin (BSA), and oleate were purchased from Sigma. Palmitate was obtained from Cayman Chemical Company. DPBS buffer without Ca^2+^ and Mg^2+^ and the Amaxa human chondrocyte nucleofector kit were purchased from Lonza. Human *CHOP*-specific small interfering RNA (siRNA) and primer pairs for *nupr1, CHOP, TRB3*, and TATA box-binding protein (*TBP*) were obtained from Qiagen. Human *nupr1*-specific siRNA, nitrocellulose membranes, and ECL Western blotting detection reagents were purchased from GE Healthcare. iTaq Universal SYBR Green Supermix was obtained from Bio-Rad Laboratories. Antibodies to Nupr1 and TRB3, and TUNEL assay kit were purchased from Abcam. Cell lysis buffer, antibodies to CHOP, cytochrome *c*, cleaved caspase-3 (CC3), activating transcription factor 4 (ATF4), phosphorylated Akt at Ser473 (P-Akt), total Akt (T-Akt), and glyceraldehyde 3-phosphate dehydrogenase (GAPDH) were obtained from Cell Signaling Technology. A full-length human *nupr1* plasmid (pcDNA3-*nupr1*) construct was kindly provided by Dr Juan L. Iovanna (Marseille, France). All of custom-designed primers were purchased from Invitrogen.

### Chondrocyte isolation and culture conditions

Normal human cartilage tissue was obtained from the ankle joints of organ donors provided by the Gift of Hope Organ and Tissue Donor Network (Chicago, IL) through an agreement with Rush University Medical Center (Chicago, IL). Each cartilage specimen was graded for degenerative changes based on the five-point Collins scale [27]. Only cells from tissue graded as 0 or 1 (indicating no symptom of arthritis) were used in experiments. All the procedures and experimental protocols are approved by the Institutional Review Board (IRB) of the Wake Forest School of Medicine. The authors did not have access to any identifiable information and, as per the IRB that approved the study, no further consent was required. The ages of tissue donors ranged from 31 to 72 years. Chondrocytes were isolated and cultured as described [16]*.* In brief, cells were isolated under aseptic conditions by sequential enzymatic digestion at 37°C using pronase at 2 mg/ml in serum-free DMEM/F-12/antibiotics for 1 h, followed by overnight digestion with collagenase-P at 0.36 mg/ml in DMEM/F-12 (5% FBS). Viability of isolated cells was determined using trypan blue and cells were counted using a hemocytometer. Monolayer cultures were established by plating cells in six-well plates at 2 × 10^6^ cells/well in DMEM/F-12 medium supplemented with 10% FBS at 37°C and 5% CO_2_. Under these culture conditions, primary human articular chondrocytes maintain their chondrocytic phenotype (data not shown). At confluence, cultures were changed to serum-free media and cultured 6 h before use in experiments.

### Chondrocyte treatment and immunoblotting

Palmitate and oleate were conjugated to fatty acid-free BSA, as described previously [8]. Chondrocyte monolayers were changed to serum-free media/antibiotics for 6 h followed by treatments with BSA alone (as a control) or 500 μM of BSA-conjugated FFA (palmitate or oleate) at 37°C and 5% CO_2_ overnight. After treatments, cells were washed with DPBS containing 1 mM PMSF and lysed with the cell lysis buffer containing 1 mM PMSF and 1/100 dilution of phosphatase inhibitor cocktail 2. The lysates were rotated on a tube rotator at 4°C for 30 min and then centrifuged (18000×***g***, 10 min) at 4°C to remove any insoluble materials. Soluble proteins were quantitated by the Micro BCA protein assay kit. Samples containing equal amounts of total proteins were separated by sodium dodecyl sulfate-polyacrylamide gel electrophoresis and transferred to nitrocellulose for immunoblotting with anti-CHOP, cytochrome *c*, CC3, Nupr1, TRB3, ATF4, P-Akt, T-Akt and GAPDH antibodies, respectively. Immunoreactive bands were detected with the ECL Western blotting detection reagents. Densitometry was performed on immunoblots using ImageJ software (National Institute of Health). All immunoblotting experiments were repeated at least three times with similar results using cells from independent donors.

### Chondrocyte transfection

Chondrocytes were transfected by nucleofection method using the Amaxa human chondrocyte nucleofector kit. Chondrocyte monolayers were treated with DMEMF/antibiotics containing 0.2% (w/v) pronase, 0.036% (w/v) collagenase P and 10% (v/v) FBS for 3 h to detach the cells. The cells were collected by centrifugation (540×***g***, 10 min) and washed once with DPBS. They were then resuspended in the nucleofector solution containing siRNA (50 pmol/million cells) or plasmid DNA (0.5 µg/million cells), followed by transfection using program U-24 in the Amaxa Nucleofector II. After transfection, cells were immediately transferred into warmed DMEMF/antibiotics containing 20% (v/v) FBS in six-well plates previously coated with poly-lysine to help cell attachment, and incubated at 37°C and 5% CO_2_ overnight for recovery, then changed to DMEMF/antibiotics containing 10% (v/v) FBS overnight prior to cell treatment.

### RNA isolation and quantitative real-time polymerase chain reaction (qRT-PCR)

Total RNA was isolated from the treated chondrocytes by the TRIzol method as described previously [28]. To synthesize complementary DNA, isolated total RNA (0.5–1 µg) was combined with 0.5 µg of random primers (Invitrogen) in 14 µl of RNase-free water, incubated at 70°C for 5 min, then cooled immediately on ice. Two hundred units of M-MLT reverse transcriptase (Promega) and dNTP Mix (2.5 mM) were then added into the RNA solution in a total volume of 25 µl of 1× reaction buffer (Promega), and incubated at 37°C for 60 min. Equivalent amounts of complementary DNA were used for real-time PCR in a 20-µl reaction mixture containing 10 µl of iTaq universal SYBR green supermix (Bio-Rad) and 1 µl of primer pairs specific for *nupr1, CHOP, TRB3*, or *TBP* (Qiagen). The qRT-PCR was performed using a 7500 Fast Real-Time PCR System (Applied Biosystems) and analyzed using 7500 Software v2.0.5 (Applied Biosystems) by the comparative *C*_T_ method [28]. The expression level of targeted genes was normalized to *TBP* expression measured in parallel samples.

### TUNEL staining

Chondrocytes apoptosis was further confirmed by TUNEL assay [29]. Human chondrocytes were cultured, treated with BSA alone or BSA-conjugated FFA (palmitate or oleate) and were stained according to manufacturer protocol. Cells were then visualized by Echo revolve fluorescence microscope.

### Primer design for quantitation of CHOP transcript variants by qRT-PCR

Human gene database analysis from the National Center for Biotechnology Information (NCBI) showed six alternatively spliced *CHOP* transcript variants (1–6) containing accumulative deletions in the promoter region (see Figure 6A for details). To evaluate the relative abundance of each of the *CHOP* transcript variants in human chondrocytes, we diligently designed the forward primers for each variant and for total *CHOP* transcripts that contained a shared sequence (TGAAAGCAG). PCR was performed with these sets of forward primers and a common reverse primer. All primers were designed to have very similar *T*_m_ values (approximately 60°C), providing uniform PCR efficiency and amplification conditions for qRT-PCR described above. Sequences for each primer are shown in the Figure 6B.

### Statistical analysis

Data are expressed as mean ± standard deviation of at least three independent replicates. Statistical analysis was performed by the paired *t*-test with exact *P* values using Prism 7 (version 7.00). The results were considered statistically significant at a value of *P*<0.05.

## Results

### Palmitate induces expressions of Nupr1 and TRB3 in human primary articular chondrocytes

**Figure 1:**
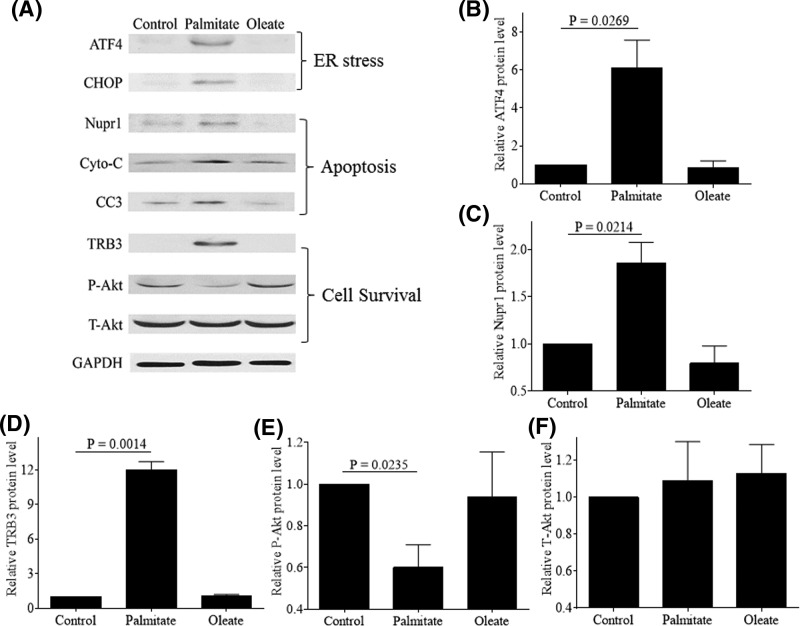
Palmitate induces ER stress and increases expressions of Nupr1 and TRB3 (**A**) Human chondrocytes were stimulated with 500 µM BSA-conjugated palmitate and oleate overnight and probed for ATF4, CHOP, Nupr1, cytochrome *c*, CC3, TRB3, P-Akt, T-Akt, and GAPDH antibodies, respectively. Blots were stripped and reprobed with GAPDH as a loading control. Densitometric analysis for protein levels of ATF4 (**B**), Nupr1 (**C**), TRB3 (**D**), P-Akt (**E**), and T-Akt (**F**) were performed on blots obtained in three independent experiments similar to the one shown in panel (A). Data were shown as mean ± standard deviation of the mean; Cyto-C, cytochrome *c*.

Saturated fatty acid palmitate is known to induce ER stress and promote apoptosis in human chondrocytes [7,9]. Hence, we wanted to further understand the molecular mechanism involved. Treatment of normal human articular chondrocytes with palmitate, but not oleate, increased the expressions of ATF4 and CHOP (Figure 1A,B and Supplementary Figure S1A) that are markers for ER stress, and cytochrome *c* as well as CC3 (Figure 1A and Supplementary Figure S1B,C), which are markers for apoptosis. Furthermore, the treatment of chondrocytes with palmitate also induced the expressions of Nupr1 and TRB3 (Figure 1A,C,D). TRB3 is a negative regulatory of Akt [17], which is pro-survival molecule. In our study, the stimulation of cells with palmitate reduced Akt phosphorylation at Ser473 (P-Akt) without altering the levels of total Akt (T-Akt) (Figure 1A,E,F). These data suggest that palmitate-induced TRB3 expression might inhibit Akt phosphorylation and negatively regulates the survival pathways in human chondrocytes. Taken together, our results imply that both Nupr1 and TRB3 are involved in palmitate-induced apoptosis in human articular chondrocytes.

### CHOP is required for palmitate-induced expressions of Nupr1 and TRB3

**Figure 2:**
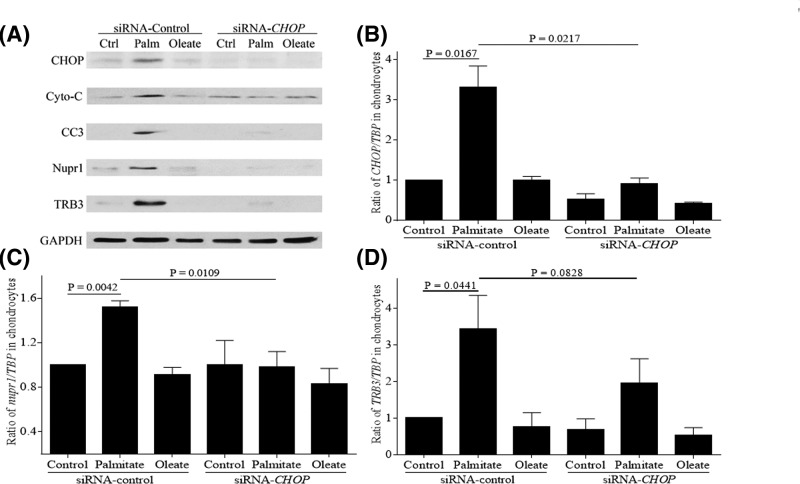
Knockdown of CHOP expression inhibits palmitate-induced increased expressions of Nupr1 and TRB3 in human chondrocytes Human chondrocytes were transfected with control siRNA or siRNA specific for *CHOP*, and then were stimulated with 500 µM BSA-conjugated palmitate and oleate overnight and probed for CHOP, cytochrome *c*, CC3, Nupr1, and TRB3 (**A**). Blots were stripped and reprobed with GAPDH as a loading control. In some experiments, total RNA was isolated, cDNA synthesized, and qRT-PCR was performed using primers specific for *CHOP* (**B**), *nupr1* (**C**), and *TRB3* (**D**). Data were normalized to TBP as a control and shown as mean ± standard deviation of the mean. Ctrl, Control; Palm, Palmitate.

Since our study showed that CHOP is induced by palmitate, we wanted to examine if CHOP regulates the expression of Nupr1 and TRB3 during palmitate-induced ER stress. Using CHOP-specific siRNA, we achieved approximately 75% reduction in CHOP protein expression (Figure 2A and Supplementary Figure S2A) and mRNA (Figure 2B). Knocking down CHOP expression reduced the palmitate-induced expression of Nupr1 and TRB3 at both protein (Figure 2A and Supplementary Figure S2D,E) and mRNA level (Figure 2C,D). Furthermore, CHOP knockdown also reduced the palmitate-induced expressions of pro-apoptotic molecules cytochrome *c* and CC3 (Figure 2A and Supplementary Figure S2B,C), and reduced apoptotic DNA fragmentation as detected by TUNEL staining (Figure 5A). Together, these results demonstrate that CHOP plays a major role in palmitate-mediated apoptosis and that CHOP is upstream of both Nupr1 and TRB3 in articular chondrocytes.

### Nupr1 plays a crucial role in palmitate-induced apoptosis

**Figure 3:**
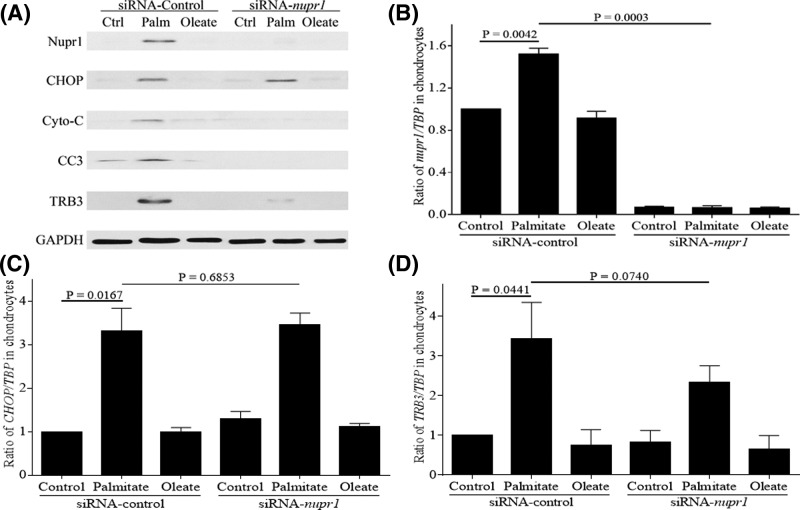
Knockdown of Nupr1 expression inhibits palmitate-induced expressions of cytochrome *c*, CC3, and TRB3, but not CHOP Human chondrocytes were transfected with control siRNA or siRNA specific for *nupr1*, and then were stimulated with 500 µM BSA-conjugated palmitate and oleate overnight and probed for Nupr1, CHOP, cytochrome *c*, CC3, and TRB3 (**A**). Blots were stripped and reprobed with GAPDH as a loading control. In some experiments, total RNA was isolated, cDNA synthesized, and qRT-PCR performed using primers specific for *nupr1* (**B**), *CHOP* (**C**), and *TRB3* (**D**). Data were normalized to TBP as a control and shown as mean ± standard deviation of the mean. Ctrl, Control; Palm, Palmitate.

To further understand the role of Nupr1 in palmitate-induced apoptosis, we knocked down Nupr1 expression by siRNA and examined its effect on expressions of CHOP, TRB3 and pro-apoptotic molecules cytochrome *c* and CC3. Using *nupr1*-specific siRNA, we achieved an approximately 95% reduction in *nupr1* mRNA (Figure 3B) and protein expressions (Figure 3A and Supplementary Figure S3A). Knocking down Nupr1 expression reduced palmitate-induced TRB3 expressions at both protein and mRNA levels (Figure 3A,D and Supplementary Figure S3E), and also decreased protein expressions of cytochrome *c* and CC3 (Figure 3A and Supplementary Figure S3C,D), but had no effect on CHOP expression at both protein and mRNA levels (Figure 3A,C and Supplementary Figure S3B). Knocking down Nupr1 also reduced apoptotic DNA fragmentation as detected by TUNEL staining (Figure 5A). These results suggest that Nupr1 is required for palmitate-induced apoptosis and that CHOP is functionally upstream of Nupr1 in human chondrocytes.

**Figure 4:**
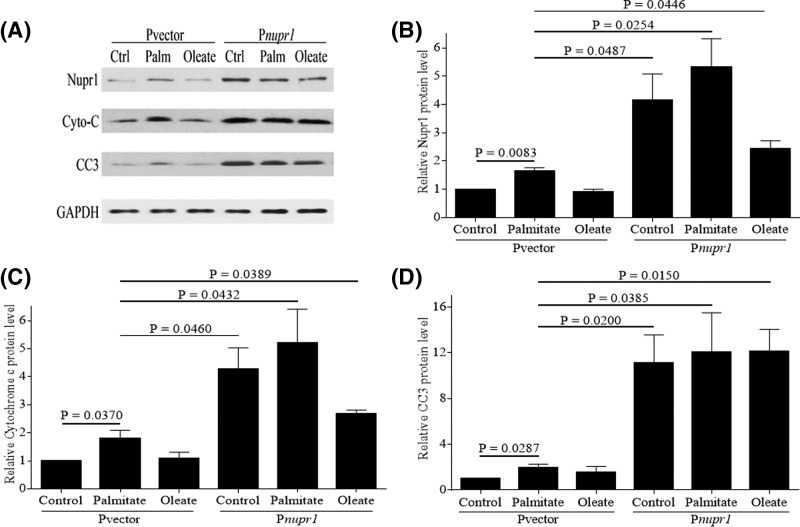
Overexpression of Nupr1 promotes caspase-mediated apoptosis in human normal chondrocytes A full-length *nupr1* plasmid construct (P*nupr1*) or empty vector (Pvector) was transfected into human chondrocytes, and then were stimulated with 500 µM BSA-conjugated palmitate and oleate overnight and probed for Nupr1, cytochrome *c* and CC3 (**A**). Blots were stripped and reprobed with GAPDH as a loading control. Densitometric analysis for protein levels of Nupr1 (**B**), cytochrome *c* (**C**) and CC3 (**D**) were performed on blots obtained in three independent experiments similar to the one shown in panel (A). Data were shown as mean ± standard deviation of the mean. Ctrl, Control; Palm, Palmitate.

**Figure 5:**
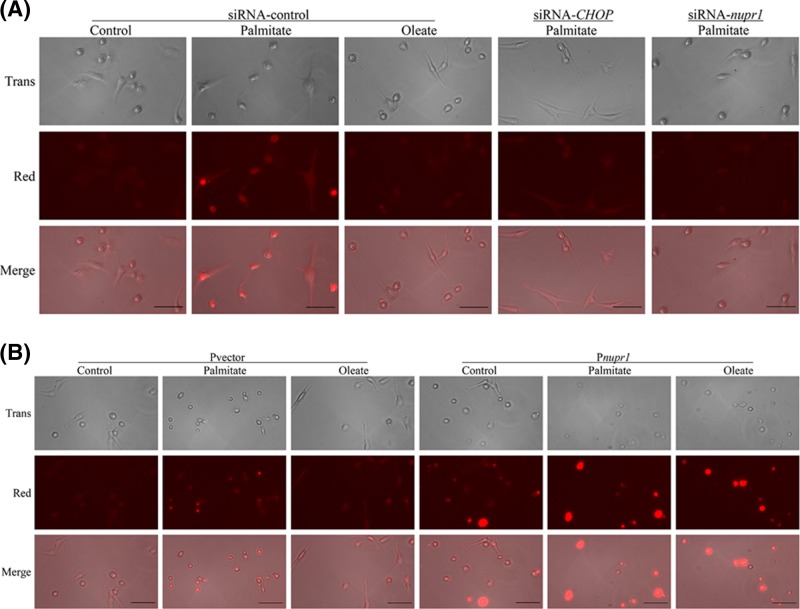
Evaluation of palmitate-induced apoptosis in human chondrocytes by TUNEL assay Human chondrocytes were transfected with siRNAs (**A**) or plasmid containing full-length of *nupr1* (**B**) and treated with 500 µM BSA-conjugated palmitate and oleate overnight, and apoptosis was assessed by TUNEL assay; scale bar, 50 µm.

Next, we wanted to examine if overexpression of Nupr1 promotes apoptosis in normal human chondrocytes. Transient transfection of a full-length human *nupr1* construct in chondrocytes increased Nupr1 expression by 3- to 4-fold (Figure 4A,B). Overexpression of Nupr1 significantly increased the basal expressions of cytochrome *c* and CC3 compared with control (Pvector) cells. Treatment with palmitate had no additional effect (Figure 4A,C,D). Overexpression of Nupr1 in chondrocytes also increased TUNEL staining (Figure 5B). Taken together, our results demonstrate that Nupr1 plays a crucial role in palmitate-induced apoptosis in human articular chondrocytes.

### Human chondrocytes predominantly express CHOP transcript variant 5

**Figure 6:**
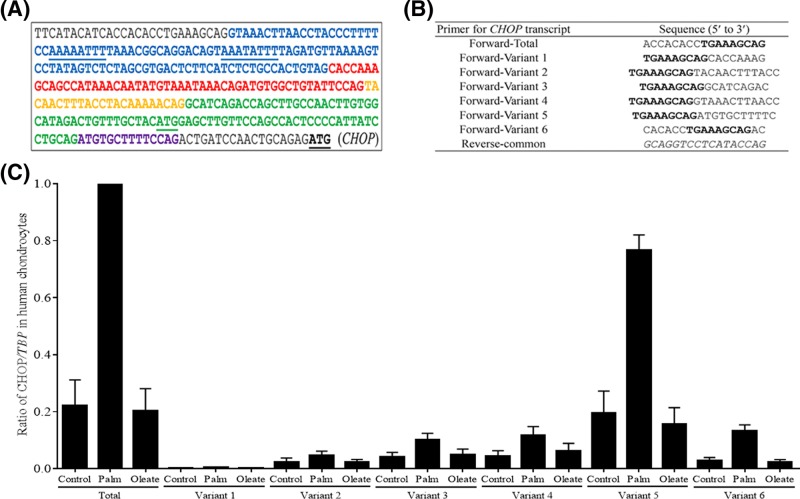
The relative abundances of individual CHOP transcript variants in human normal chondrocytes (**A**) Putative Nupr1-binding elements were only identified in the longest human *CHOP* transcript variant 4. Compared with the variant 4, variant 1 is lacking the blue region; variant 2 is lacking the blue + red regions; variant 3 is lacking the blue + red + yellow regions; variant 5 is without the blue + red + yellow + green regions; variant 6 is lacking all colored regions. *CHOP* transcript variants 1–4 are predicted to use the first starting codon (ATG) in green and variants 5–6 are predicted to use the second starting codon. (**B**) Primer design for quantitation of *CHOP* transcript variants by qRT-PCR. All of forward primers contain the shared sequences in bold. Each of forward primers were paired with a common reverse primer in italic. (**C**) Abundances of *CHOP* transcript variants 1–6 relative to total *CHOP* transcripts. Human chondrocytes were stimulated with 500 µM BSA-conjugated palmitate and oleate overnight, total RNA was isolated, cDNA synthesized, and qRT-PCR performed using primer pairs specific for individual *CHOP* transcript variants or total *CHOP* transcripts in panel (B). Data were normalized to TBP as a control, expressed as a ratio relative to total *CHOP* transcripts by palmitate treatment, and shown as mean ± standard deviation of the mean. Palm, Palmitate.

Our results indicated that CHOP is upstream of Nupr1 in human chondrocytes, which is in contrast with earlier reports [23,24,30]. NCBI human gene database analysis revealed six variants of CHOP transcript that are expressed by various cell types; however, only variant 4, the longest CHOP transcript, has the putative Nupr1-binding elements [31–33] (Figure 6A). Hence, we wanted to determine which variant(s) of CHOP is expressed by human chondrocytes. Our specially designed primer pairs (Figure 6B) detected all six CHOP transcript variants in chondrocytes in response to palmitate treatment albeit with different abundances (Figure 6C). Variant 5 was found to be most dominant/abundant CHOP transcript accounting for approximately 70% of total CHOP transcripts in human chondrocytes (Figure 6C).

## Discussion

**Figure 7:**
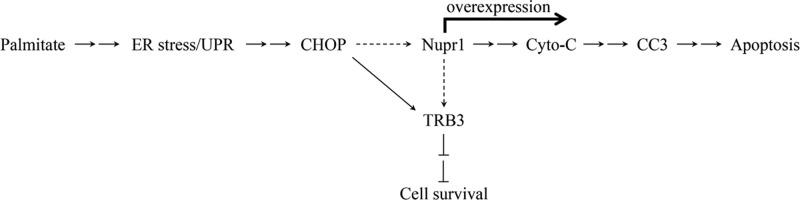
Model showing the CHOP/ Nupr1/TRB3 signaling nexus in palmitate-ER stress induced apoptosis in human chondrocytes Palmitate induces ER stress and activation of the UPR pathway to stimulate CHOP expression, leading to cytochrome *c* release and activation of caspase-mediated apoptotic pathways via increasing Nupr1 expression. Both CHOP and Nupr1 up-regulate TRB3 expression to modulate cell survival. Nupr1 overexpression markedly promotes caspase-mediated apoptosis. ER, endoplasmic reticulum; UPR, unfolded protein response; Cyto-C, cytochrome *c*; CC3, cleaved caspase-3; →, single-step stimulation; ----, putative single-step stimulation; → →, multistep stimulations; ⊥ ⊥, multistep inhibitions.

Emerging evidence suggest that apoptosis plays a key role in OA pathology [34]. However, the underlying mechanisms are not clearly understood. In the present study, we found that the saturated FFA palmitate activate apoptotic pathways in articular chondrocytes via increased expressions of CHOP, Nupr1 and TRB3. CHOP and Nupr1 are transcription factors that up-regulate apoptotic molecules and pathways. Whereas TRB3, a member of the *tribbles* pseudo kinase family, is known to negatively regulate cell survival pathways. Our data demonstrated that CHOP is key regulator of both Nupr1 and TRB3 and that the Nupr1 expression is regulated by CHOP in human chondrocytes. Knocking down the expression of either CHOP or Nupr1 decreased palmitate induced expressions of downstream TRB3, cytochrome *c* and CC3. Likewise, overexpression of Nupr1 up-regulated expressions of pro-apoptotic molecules cytochrome *c*, and CC3, and promoted apoptosis. Taken together, our data clearly suggest that Nupr1 plays a critical role in conjunction with CHOP and TRB3 in regulating the palmitate-induced apoptosis in human primary chondrocytes (Figure 7). To the best of our knowledge, this is the first study to demonstrate the role of Nupr1 in palmitate-induced cell death and as a possible mechanism for obesity-linked OA.

Saturated fatty acid palmitate has been shown to induce ER stress in chondrocytes and meniscus cells [8,9]. Sustained and unresolved ER stress promotes cell death via two pathways: the Ire1/TRAF2/ASK pathway and PERK/ATF6/CHOP axis [35]. Interaction of TRAF2 with Ire1 activates an apoptosis signal-regulating kinase 1 (ASK1), a mitogen-activated protein kinase kinase kinase (MAPKKK) that activates JNK. Activation of JNK promotes apoptosis via the caspase system [36]. We have previously shown that treatment of chondrocytes with palmitate activated JNK signaling pathway in chondrocytes [10] and the blocking Ire1 signaling pathway inhibited palmitate-ER stress induced apoptosis in meniscus cells [8]. CHOP is a key transcriptional factor and pro-apoptotic molecule that is induced during ER stress and is thought to mediate apoptosis via suppressing the expression of Bcl-2, a pro-survival protein [37]. Overexpression of CHOP has been shown to promote apoptosis in chondrocytes [38]. In the present study, palmitate treatment of normal human articular chondrocytes increased the expression of CHOP and activated apoptotic pathways. Knocking down CHOP expression inhibited apoptosis and reduced the expression of Nupr1 and TRB3, the two known negative regulators of cell survival [12,13].

Interestingly, Nupr1 is known to regulate both CHOP and TRB3 in human tumor cells [23] and neuronal cells [24]. However, in our study knocking down Nupr1 decreased the expression of TRB3 but not CHOP, demonstrating that CHOP is functional upstream of Nupr1 in human chondrocytes, which is in contrast with previously published reports [23,24,30]. Nupr1 is thought to bind to an AT-rich element via its AT-hook similar to HMGA1α [31–33]. In support of our results, NCBI human gene database analysis of the TRB3 promoter showed multiple known CHOP-binding sites [13,26] and a putative binding site for Nupr1. Similar Nupr1-binding elements were found on CHOP promoter region also; however, these elements were present only in the longest human CHOP transcript variant 4. There are six known splice variants of CHOP transcripts that are expressed by various cells (http://www.ensembl.org). Our study showed that human chondrocytes express predominantly variant 5 that lacks putative Nupr1-binding site. Thus, the type of CHOP transcript variants expressed in human cells might be different depending upon the cell type and relative amount of stress present [26,38]. Interestingly, analysis of *nupr1* promoter region revealed a putative CHOP-binding site, which supports our data of CHOP being upstream of Nupr1 in human chondrocytes.

ER stress activates the mitochondrial apoptotic pathway, resulting in the release of cytochrome *c* from the mitochondria [39]. How Nupr1 regulates cytochrome *c* expression/release in palmitate-induced apoptosis is poorly understood. Since the members of Bcl-2 family have been found to trigger mitochondrial outer membrane permeability to release cytochrome *c* [39,40], it is possible that Nupr1 might increase cytochrome *c* expression/release by regulating the Bcl-2 family member(s). Interestingly, putative Nupr1-binding sites have been detected in multiple members of Bcl-2 family. The potential interaction between Nupr1 and Bcl-2 family members and its role in palmitate-induced apoptosis in chondrocytes needs further investigation.

TRB3 is a pseudo-kinase that is up-regulated during ER stress and is a negative regulator of Akt [23]. Increased expression of TRB3 decreases phosphorylation of Akt at serine 473 and threonine 308 [17]. The Akt signaling pathway is crucial for cell survival. Activation of the Akt pathway blocks the apoptotic pathway by negatively regulating the activity of BAD, a member of the Bcl2 family of proteins that binds to Bcl2 or Bcl-X_L_ and inhibits their anti-apoptotic activity [41]. Akt regulates the ASK1/JNK mediated pro-apoptotic pathway via phosphorylation of ASK1 at ser83, which inhibits ASK1 activity [42]. In the present study, treatment of chondrocytes with palmitate increased the expression of TRB3 and decreased phosphorylation of Akt at ser473. In our earlier studies, we have shown that palmitate induced activation of the ASK1/JNK pathway and that blocking JNK signaling pathway restored phosphorylation of Akt at ser473 [8,9]. Taken together, these studies show that TRB3 in conjunction with CHOP and Nupr1 plays an important role in palmitate-mediated apoptosis.

In conclusion, our results demonstrated that Nupr1 play a critical role in palmitate-induced apoptosis in human chondrocytes. To the best of our knowledge, this is first study to show a nexus between CHOP, Nupr1, and TRB3 in regulating palmitate-induced apoptosis in human chondrocytes that could play an important role in obesity-linked OA.

## Supporting information

**Figure S1:** Palmitate induces ER stress and increases expressions of CHOP, cytochrome c and CC3. Human chondrocytes were stimulated with 500 μM BSA-conjugated palmitate and oleate overnight and probed for CHOP, cytochrome c and CC3 antibodies, respectively. Blots were stripped and reprobed with GAPDH as a loading control. Densitometric analysis for protein levels of CHOP (A), cytochrome c (B) and CC3 (C) were performed on blots obtained in three independent experiments similar to the one shown in Figure 1A. Data were shown as mean ± standard deviation of the mean.

**Figure S2:** Knock down of CHOP expression inhibits palmitate-induced increased expressions of cytochrome c, CC3, Nupr1 and TRB3 in human chondrocytes. Human chondrocytes were transfected with control siRNA or siRNA specific for *CHOP*, and then were stimulated with 500 μM BSA-conjugated palmitate and oleate overnight and probed for CHOP, cytochrome C, CC3, Nupr1 and TRB3. Blots were stripped and reprobed with GAPDH as a loading control. Densitometric analysis for protein levels of CHOP (A), cytochrome c (B), CC3 (C), Nupr1 (D) and TRB3 (E) were performed on blots obtained in three independent experiments similar to the one shown in Figure 2A. Data were shown as mean ± standard deviation of the mean.

**Figure S3:** Knock down of Nupr1 expression inhibits palmitate-induced expressions of cytochrome c, CC3 and TRB3, but not CHOP. Human chondrocytes were transfected with control siRNA or siRNA specific for *nupr1*, and then were stimulated with 500 μM BSA-conjugated palmitate and oleate overnight and probed for Nupr1, CHOP, cytochrome c, CC3 and TRB3. Blots were stripped and reprobed with GAPDH as a loading control. Densitometric analysis for protein levels of Nupr1 (A), CHOP (B), cytochrome c (C), CC3 (D) and TRB3 (E) were performed on blots obtained in three independent experiments similar to the one shown in Figure 3A. Data were shown as mean ± standard deviation of the mean.

## Abbreviations

AKT: protein kinase B/AKT
CC3: cleaved caspase -3
CHOP: CCAAT-enhancer-binding protein homologous protein
DMEMF: Dulbecco’s modified Eagle’s medium /Ham’s F-12
ER stress: endoplasmic reticulum stress
FBS: fetal bovine serum
FFA: free fatty acid
Nupr1: nuclear protein 1
OA: osteoarthritis
PMSF: phenylmethanesulfonyl fluoride solution
TRB3: *tribbles* homolog related protein 3
