# Supplementary material for: Nupr1 regulates palmitate-induced apoptosis in human articular chondrocytes
Source: Biosci Rep. 2019 Feb 15;39(2):BSR20181473. doi: 10.1042/BSR20181473 (PMC6379229; doi:10.1042/BSR20181473)
Supplement: Supplementary file 1 [file bsr-39-bsr20181473_Supp1.pdf]

Figure legends for Supplementary Material

Figure S1

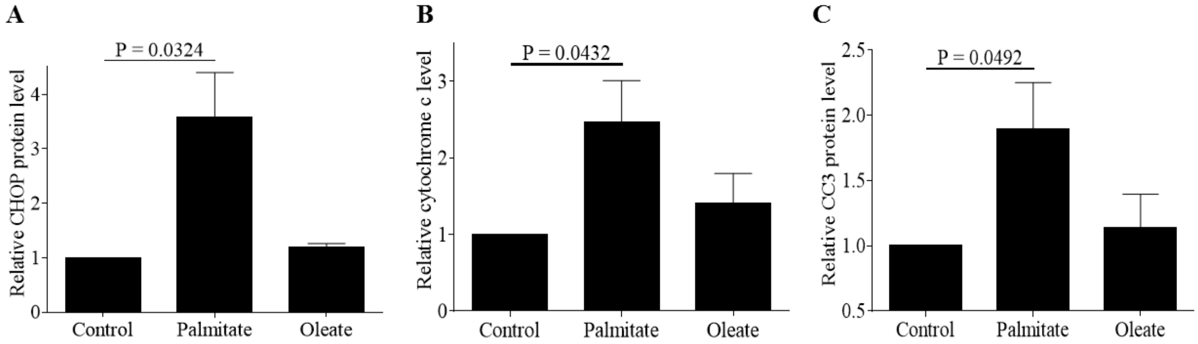

Figure S1. Palmitate induces ER stress and increases expressions of CHOP, cytochrome c and CC3. Human chondrocytes were stimulated with 500 µM BSA-conjugated palmitate and oleate overnight and probed for CHOP, cytochrome c and CC3 antibodies, respectively. Blots were stripped and reprobed with GAPDH as a loading control. Densitometric analysis for protein levels of CHOP (A), cytochrome c (B) and CC3 (C) were performed on blots obtained in three independent experiments similar to the one shown in Figure 1A. Data were shown as mean ± standard deviation of the mean.

Figure S2

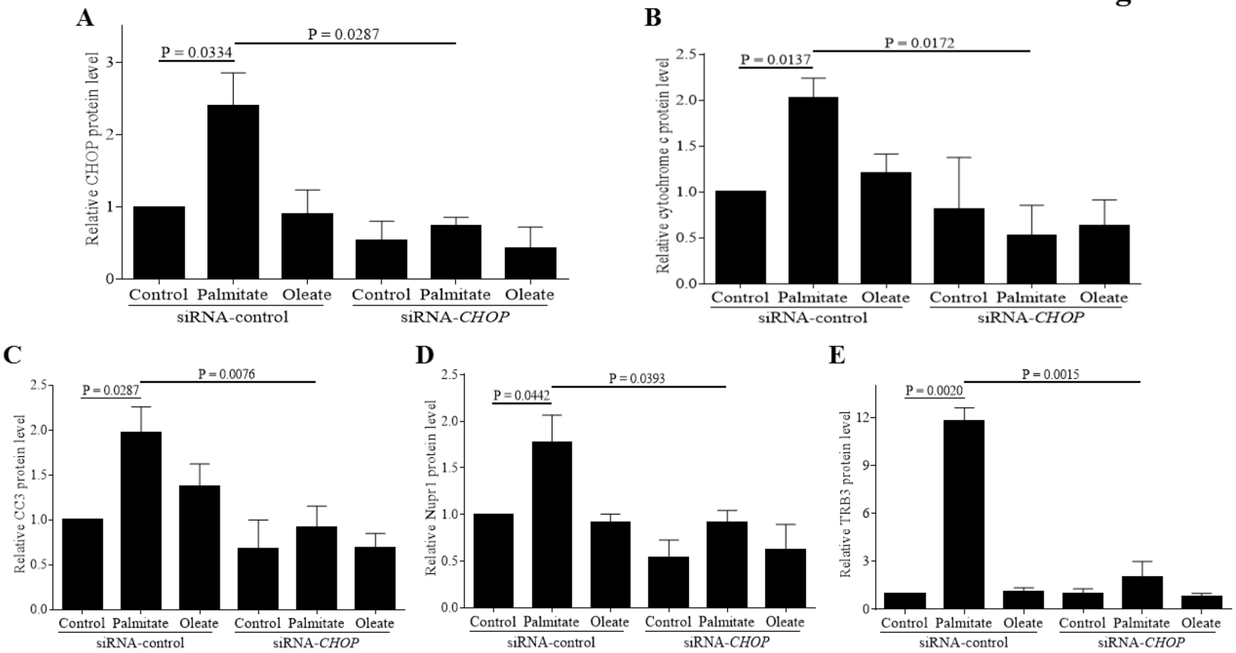

Figure S2. Knock down of CHOP expression inhibits palmitate-induced increased expressions of cytochrome c, CC3, Nupr1 and TRB3 in human chondrocytes. Human chondrocytes were transfected with control siRNA or siRNA specific for *CHOP*, and then were stimulated with 500 μM BSA-conjugated palmitate and oleate overnight and probed for CHOP, cytochrome C, CC3, Nupr1 and TRB3. Blots were stripped and reprobed with GAPDH as a loading control. Densitometric analysis for protein levels of CHOP (A), cytochrome c (B), CC3 (C), Nupr1 (D) and TRB3 (E) were performed on blots obtained in three independent experiments similar to the one shown in Figure 2A. Data were shown as mean ± standard deviation of the mean.

Figure S3

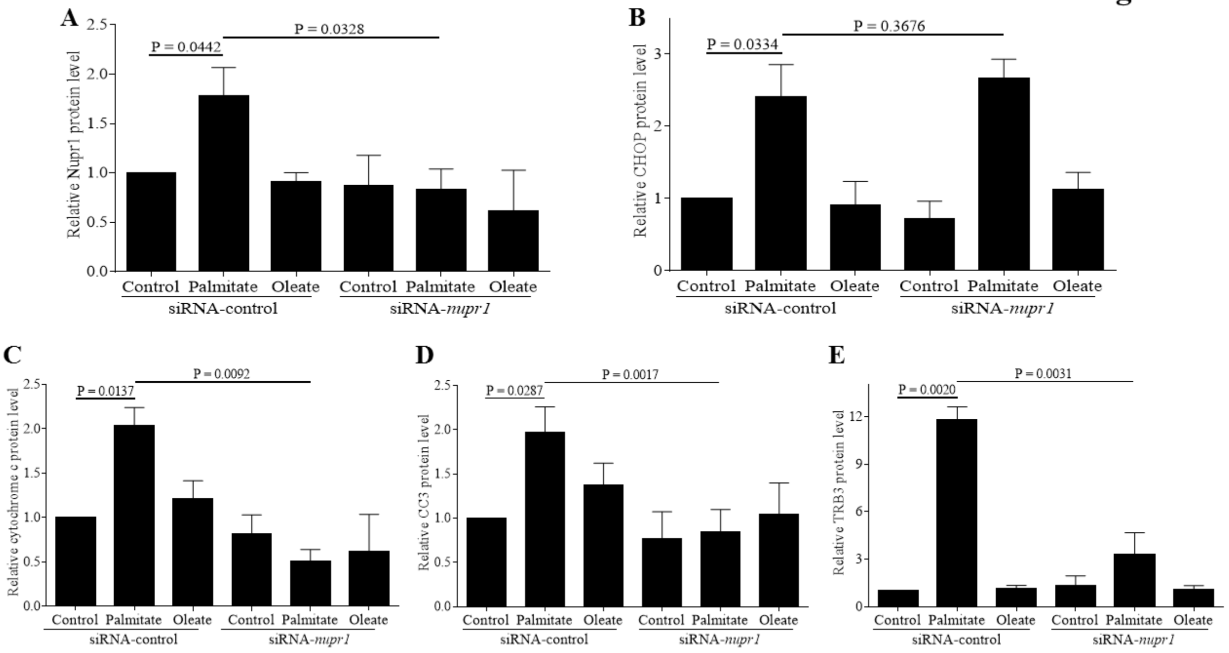

Figure S3. Knock down of Nupr1 expression inhibits palmitate-induced expressions of cytochrome c, CC3 and TRB3, but not CHOP. Human chondrocytes were transfected with control siRNA or siRNA specific for *nupr1*, and then were stimulated with 500 µM BSA-conjugated palmitate and oleate overnight and probed for Nupr1, CHOP, cytochrome c, CC3 and TRB3. Blots were stripped and reprobed with GAPDH as a loading control. Densitometric analysis for protein levels of Nupr1 (A), CHOP (B), cytochrome c (C), CC3 (D) and TRB3 (E) were performed on blots obtained in three independent experiments similar to the one shown in Figure 3A. Data were shown as mean ± standard deviation of the mean.
